# The TAO-Gen Algorithm for Identifying Gene Interaction Networks with Application to SOS Repair in *E. coli*

**DOI:** 10.1289/txg.7105

**Published:** 2004-07-21

**Authors:** Takeharu Yamanaka, Hiroyoshi Toyoshiba, Hideko Sone, Frederick M. Parham, Christopher J. Portier

**Affiliations:** ^1^Laboratory of Computational Biology and Risk Analysis, National Institute of Environmental Health Sciences, National Institutes of Health, Department of Health and Human Services, Research Triangle Park, North Carolina, USA

**Keywords:** gene networks, microarray, Bayesian model selection, SOS repair, toxicogenomics

## Abstract

One major unresolved issue in the analysis of gene expression data is the identification and quantification of gene regulatory networks. Several methods have been proposed for identifying gene regulatory networks, but these methods predominantly focus on the use of multiple pairwise comparisons to identify the network structure. In this article, we describe a method for analyzing gene expression data to determine a regulatory structure consistent with an observed set of expression profiles. Unlike other methods this method goes beyond pairwise evaluations by using likelihood-based statistical methods to obtain the network that is most consistent with the complete data set. The proposed algorithm performs accurately for moderate-sized networks with most errors being minor additions of linkages. However, the analysis also indicates that sample sizes may need to be increased to uniquely identify even moderate-sized networks. The method is used to evaluate interactions between genes in the SOS signaling pathway in *Escherichia coli* using gene expression data where each gene in the network is over-expressed using plasmids inserts.

Gene expression microarrays (gene chips) have revolutionized biology by generating vast amounts of data roughly quantifying the level of mRNA expression for thousands of genes in a single sample. The analysis of these data is extraordinarily complex, resulting in a shift in biology from predominantly qualitative evaluations to quantitative approaches. With microarray technologies, scientists are forming global views of the structural and dynamic changes in genome activity during different phases in a cell’s development and following exposure to external stimulants such as environmental agents or growth factors. These views describe the molecular working of a complex information processing system: the living cell. Numerous methods have already been proposed for the analysis of gene expression data. The most commonly used methods rely on clustering (Eisen et al. 1995; Tamayo et al. 1999), significance testing (Kerr et al. 2000) and sequence motif identification (Pilpel et al. 2001). These methods do not readily reproduce gene expression networks but are more focused on the fundamental linkage between pairs of genes. Other investigators have proposed methods to identify gene regulatory networks using Boolean networks (Akutsu et al. 2000) where each gene has one of only two states (on and off), regression methods (Gardner et al. 2003), Bayesian network models (Friedman et al. 2000; Hartemink et al. 2002) and other methods (Johnson et al. 2004).

The use of genomics data in the evaluation of health hazards and risks has received considerable attention focusing on priority setting (Pesch et al. 2004), biomarker identification (Toraason et al. 2004), hazard identification (Suter et al. 2004), and dose–response analysis (Schonwalder and Olden 2003; Simmons and Portier 2002; Waters et al. 2003). If genomics is to play a direct role in dose–response assessment, there will be a need for methods that provide a direct, quantitative assessment of changes in gene expression as a function of dose and changes in toxicity as a function of changes in gene expression. Developing and modeling gene interaction networks can be quantitative and provide direct dose–response data for use in risk assessment. They also are an excellent means of identifying agents that provide identical changes in expression across a broad spectrum of genes and help link agents on the basis of similar mechanistic changes.

Bayesian networks are well suited for inferring genetic interactions because of their ability to model causal influence between genes linked as a network and because they are an effective method for modeling the joint density of all variables in a system. However, the approaches suggested to date have generally focused on conversion of gene expression data to discrete states and have avoided the use of formal statistical methods for quantifying the joint density of the resulting parameters.

In this article we describe a method for inferring an “optimal” gene interaction network from microarray-based gene expression data. Unlike other network identification methods, the analytical approach presented here uses the actual measured observations on gene expression (rather than discretized data) and incorporates prior distributions for all parameters in the gene interaction network model. The method encompasses model selection theory from Bayesian regression to find gene network structures suitable for given data sets. Computer simulations presented in this article demonstrate that the proposed method is capable of identifying networks, given the sample size is sufficiently large. For small networks the limited number of replicates used for most microarray studies available today are adequate; for larger networks other options are discussed.

## Materials and Methods

**Figure 1:**
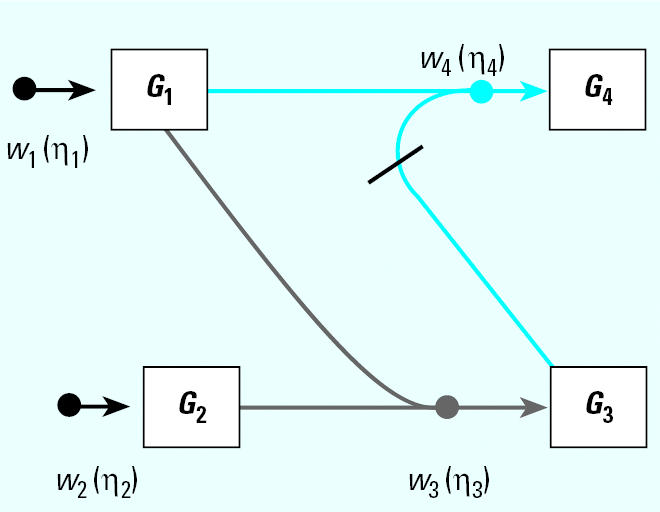
A simple gene interaction network consisting of four genes.

Figure 1 illustrates the general structure of a four gene regulatory system where the linkage between expression of gene *i* and expression of its parents (indirect regulators to gene *i*) is described by weighting the function *w_i_* (η*_i_*), where the subscript *i* denotes that this weighting function pertains to the control of gene *i* expression by all genes linked to it and η*_i_* denotes the vector of parameters defining the functional relationship*.* Let *N* be a directed acyclic graph which consists of *p* vertices (genes). Each edge is also assumed to include information about the linkage between genes (i.e., activation, as in the case for the linkage between expression of gene 1 and expression of gene 4, or suppression, expression of genes 3 and 4). In essence, *N* is a discrete random variable that takes on any of the different acyclic network structures that are possible for a set of *p* genes. Define *X_i_* to be the random variable corresponding to the measured relative level of gene expression (the expression level of a target gene for an “exposed” group to the expression level of the same gene in a “control” group) for gene *G_i_*, 1 ≤ *i* ≤ *p*. For a given network, *N* = *n*, and for each *X_i_*, define the conditional density function, *f_Xi_*(*X_i_*|*pa_n_*(*X_i_*), η*_i_*) where *pa_n_*(*X_i_*) denotes the set of vertices corresponding to the parents of expression for gene *i* in the network *n* with parameters η*_i_*. All networks in the support space for *N* are assumed to satisfy the Markov property where expression of gene *i* is independent of all genes not included in *pa_n_*(*X_i_*). Application of the Markov property and imposition of the acyclic restriction allow decomposition of the joint density function into


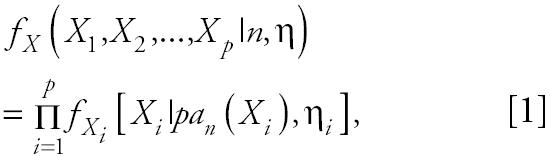


where η *=* (η_1_*,* η_2_*, . . .* η*_p_*) is the set of all parameters in the network.

Gene expression data, for the purposes of this analysis, can be expressed as a *p* by *m* matrix of the form *x* = [*x_ik_*]*_i_*_= 1,2,…_*_p_*_,_ *_k_*_= 1,2…_*_m_* where *m* is the number of observations (samples analyzed for gene expression) taken for each gene and *x**_i_* = [*x_ik_*]*_k_*_= 1,2 …_ *_m_* is the vector of all observations of expression for gene *i*. The observed gene expression levels for the parent set for gene *i* in vector notation is *pa_n_*(*x_i_*) = [*x_ijk_*]*_j_*_= 1,2,…_ *_pi_*_,_ *_k_*_=1,2 …_ *_m_* where *p_i_* is the number of parents for gene *i*. Similarly define the random vector *X*. Then, conditional on the parameters and the model, the likelihood of the data, *x**,* is given by


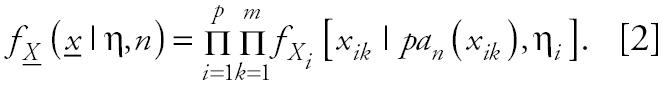


The goal of our analysis is the identification of the “best” network structure using gene expression data. Our criterion for the best network is defined as the network, n*, from the set of all acyclic networks that maximizes the posterior likelihood of the network,


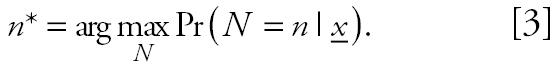


The posterior probability Pr(*N = n|**x*) is given by


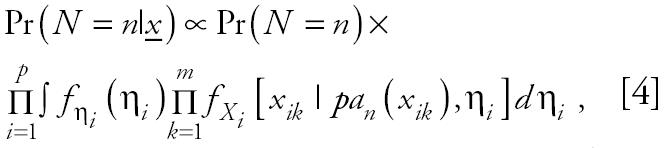


where Pr(*N = n*) and *f*_η_*_i_*(η*_i_*) are derived from the prior distributions of *N* and η*_i_* respectively, and the η*_i_* are assumed independent.

Several methods are available for assigning prior information to the distribution of countable networks for a given set of genes. One approach, which is used here, is to assume no prior knowledge by choosing *N* to be uniformly distributed (equal probability) over the space of all possible acyclic networks. By this assumption the solution to Equation 3 is identical to finding the maximum of the log of the product term in Equation 4 over the parameter space; that is the solution to Equation 3 is identical to


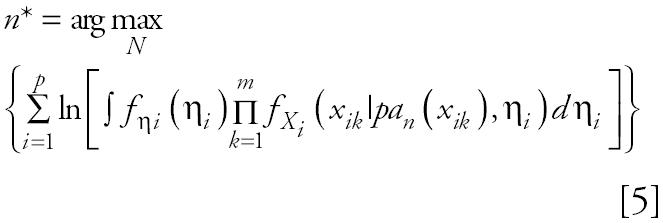


This equation is similar to the maximum likelihood estimator in classical statistical theory, but weighted over the prior densities for the parameters in the model. A clear benefit of this approach is that one does not need to estimate the model parameters while finding the best network because the integration removes those parameters from the final solution. A possible criticism of this approach is that the assumption of a uniform prior for network structure fails to completely exploit the prior knowledge of which networks are of greatest interest. This is most certainly true, but in light of our limited understanding of gene interaction networks, this appears to be a reasonable choice for a first step in network identification. When available, prior knowledge can be incorporated into this algorithm or modified algorithms to limit the space of networks to be searched; this is the solution to a different problem and will be discussed in a subsequent report.

Many possible weighting functions *w_i_*(η*_i_*) can be used to relate the relative level of expression of gene *i* to the relative levels of expression of its parents. The analysis presented here uses a log-linear model


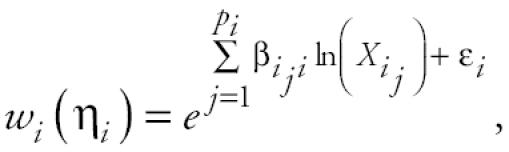


where the notation *i_j_* refers to the *j*^th^ parent of gene *i* β*_i_* = [β*_iji_*]_1×_*_pi,_* and ɛ*_i_* is a random variable with mean 0. From a mechanistic basis, using a model linear in the logarithms of the expression levels is equivalent to approximating the full nonlinear system by equations in power-law form (Kikuchi et al. 2003; Voit and Radivoyevitch 2000).

Given prior distributions for the ɛ’s and the β’s for all genes, the Markov-Chain Monte-Carlo (MCMC) method developed by Hastings (Hastings 1970) makes it possible to estimate a solution to Equation 5 and identify the “best” network. It is possible, under further restrictions, to obtain a closed form solution to the argument in Equation 5. The advantage of this approach in the framework of this article is that the entire network space can be searched exhaustively to find the best network for small networks like the ones in our simulation studies.

As is common in Bayesian linear regression theory (Gelman et al. 1995), we assume that ɛ*_i_|*σ*_i_*^2^ ~ *Normal*(0*,* σ*_i_*^2^), β*_i_|*σ*_i_*^2^ ~ *Normal*(*b**_i_*, σ*_i_*^2^*A_i_*^−1^) and σ*_i_*^2^ ~ *Gamma*(*v*_0_/2, *v*_1_/2), *v*_0_, *v*_1_ ≈ 0. These priors do not assume additional or specific information (in Bayesian parlance these are uninformative priors) and thus would be applicable for many cases. Simple algebra then results in:


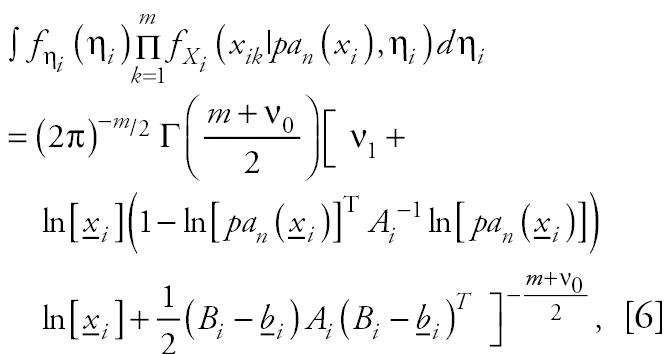


where Γ is the gamma function, *A_i_* = ln[*pa_n_*(*x**_i_*)]ln[*pa_n_*(*x**_i_*)]*^T^* and *B_i_* = ln[*x**_i_*]ln[*pa_n_*(*x**_i_*)]*^T^* *A_i_*^−1^. Given *N* = *n*, this equation allows for the direct calculation of Pr(*N* = *n|**x*). This formula is specific to these priors, but similar formulae might be derived for other cases.

Any single gene in a *p* = 4 gene network has 8 possible sets of parents (no parents, 3 single parents, 3 double parents, all other genes), hence the total number of networks including cyclic networks would be 8^4^ = 4,096 networks of which 543 are acyclic. As *p* increases, the total number of networks increases as the squared power of *p*(2*^p^*^(^*^p^*^−1)^) resulting in a very large network space to evaluate for larger networks (e.g., ~ 4 × 10^469^ for a 40-gene network). Many different types of searching algorithm could be used to limit the number of networks to be evaluated for Equation 6; through trial and error, the following modified simulated annealing algorithm (Press et al. 1989) appears to work. We will refer to this method as the TAO-Gen (Theoretical Algorithm for identifying Optimal GENe interaction networks) algorithm.

The TAO-Gen algorithm has 7 basic steps:

1. Search conditions: Restrict to ζ< *p*, the maximum number of parents for any one gene and calculate the value of Equation 6 for all ∑^ζ^*_i_*_=0_ *_p_*_−1_*C_i_* parent combinations, where *_p_C_i_* is the binomial coefficient (When *p* is relatively small, ζ = *p*−1 can be chosen and the entire network space is evaluated in this step. When *p* is even moderately large (> 10), assuming ζ = 4 or 5 will substantially reduce the computational burden). Specify a number *t* (0 ≤ *t* ≤ 1) governing the probability of local versus global switching in step 4 (*t* = 0 implies only global switching, *t* = 1 implies only local switching).
2. For the initial step *k* = 0, randomly select an order in which genes enter the network *G_k_* = (*G_k_*_1_ *G_k_*_2_ *G_k_*_3_*G_kp_*) and build a starting network choosing the parents for each gene that maximize Equation 6 while keeping the network acyclic (i.e., choose the parents for *G_k_*_1_ that are optimal first, then parents for *G_k_*_2_ that are optimal, etc.)
3. Calculate the posterior likelihood (Equation 4) for this network and denote it *L_k_*.
4. Generate a uniform random number *u*_1_ ε *uniform* (0,1) to determine the type of permutation. if *u*_1_ < *t*, the permutation occurs between two randomly chosen genes, *j* and *l*, switching the two genes for the next permutation *G_k_*_+1_,*_j_* = *G_k_*_,_*_l_* and *G_k_*_+1_,*_l_* = *G_k_*_,_*_j_*). Otherwise, make the second half of the set of genes, starting from randomly chosen gene *j*, appear first in the order (*G_k_*_|1,1_ = *G_k_*_,_*_j_*_|1_, *G_k_*_|1,2_ = *G_k_*_,_*_j_*_|2,…,_ *G_k_*_+1,_*_m_*_−_*_j_*_+1_ = *G_k_*_,1,…,_ *G_k_*_+1,_*_m_* = *G_k_*_,_*_j_*). Thus form a new gene order, *G_k_*_+1_.
5. Calculate a new posterior likelihood of the network *L_k+_*_1_ associated with the order G*_k_*_+ 1_ , as in steps 2 and 3. If L*_k_*_+1_ > L*_k_*, then keep G*_k_*_+1_. Otherwise generate a uniform random number *u*_2_ ε *uniform* (0,1)and if *u*_2_ ≤ L*_k_*_+1_/L*_k_*, keep G*_k_*_+1_ else set G*_k_*_+1_ = G*_k_*.
6. Return to step 4 and iterate.
7. Choose the network with the highest posterior probability from the sequence (*G*_0_, *G*_1_,…).

This algorithm combines aspects of the Metropolis algorithm used for Markov-Chain Monte-Carlo sampling (Hastings 1970), with the simulated annealing algorithm used for optimization (Press et al. 1989). In essence it represents a new form of genetic algorithm aimed at networks in which mutations occur in each cycle as either base-pair switches or large translocations. It may be possible under certain fixed conditions to analytically determine the degree to which the TAO-Gen algorithm reduces the number of networks to be evaluated and the efficiency with which it finds the correct solution. This is left as a separate exercise; instead, simulation studies were used to address these issues as discussed in “Results.”

### Gene Expression Data Set

Gardner et al. (2003) developed a gene-regulatory network for a nine-gene subnetwork of the SOS pathway in *Escherichia coli*. The nine genes (all gene names and locators, in parentheses following gene name, are from the EcoGene database (http://bmb.med.miami.edu/EcoGene/EcoWeb) they focused on were the principal mediators of the SOS response, *recA* (recombinase gene A, locator EC10823) and *lexA* (lambda excision gene A, locator EC10533); genes with known involvement in the SOS response, *ssb* (single strand binding gene, locator EC10976)*, recF* (recombinase gene F, locator EC10828)*, dinI* (damage inducible gene I, locator EC12670)*, umuDC* (UV mutator gene, locator EC11057); and three sigma factor genes whose function in SOS response is not clearly identified, *rpoD* (RNA polymerase factor subunit D, locator EC10896)*, rpoH* (RNA polymerase factor subunit H, locator EC10897)*,* and *rpoS* (RNA polymerase factor subunit S, locator EC10510). To quantify the subnetwork, they applied a set of nine transcriptional perturbations to *E. coli* cells in which each perturbation overexpressed a different one of the nine genes in the SOS network. Using an arabinose-controlled episomal expression plasmid, they grew the cells in batch cultures for 5.5 hr after the addition of arabinose, then measured relative change in message for their nine target genes using quantitative real-time polymerase chain reaction. In addition to the nine perturbed cultures, they also produced two additional cultures, one in which a double plasmid (*lexA*/*recA*) was incorporated into the cells and another in which 0.75 μg/mL of mitomycin C (MMC) was added to the culture to stimulate gene expression of *recA*. The resulting data set with 11 samples of relative changes in gene expression for the nine target genes is given in Table S1 in Gardner et al. (2003). In addition to the nine target genes, the nine plasmid constructs were added to the modeling as fixed stimulators of each of their respective genes to mimic changes in gene expression induced by insertion of the ten plasmid constructs. A separate stimulation by MMC was also included but with links to all genes in the network to determine if the predominant linkage to *recA* assumed by Gardner et al. (2003) was evident in the data. The exact model linking genes for sample *k* (*k* = 1,2, …11) is given by


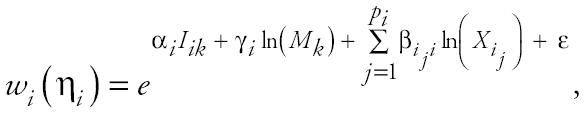


where β*_i_j_i_* is as described previously, *I_ik_* is an indicator variable equal to 1 if gene *i* has an inserted plasmid in sample *k* and is equal to 0 otherwise, α*_i_* is the magnitude of increase in gene expression induced in the *i*^th^ gene by the plasmid when it is present, *M_k_* is the relative change (relative to the standard of 0.5 μg/mL) in MMC exposure for sample *k*, and γ*_i_* is the magnitude of change in gene expression for gene *i* as a function of the relative change in MMC.

### Simulation Results

Data were simulated for a given network by sampling from the assumed error distributions and priors for a given model situation. To simulate a network, genes highest on the parental list were simulated first and the simulated values were used to simulate daughters, etc. Different starting points and different priors were used to estimate parameters in both the simulated data and the SOS data; these had no impact on the final results provided the priors chosen were uninformative.

## Results

**Figure 2:**
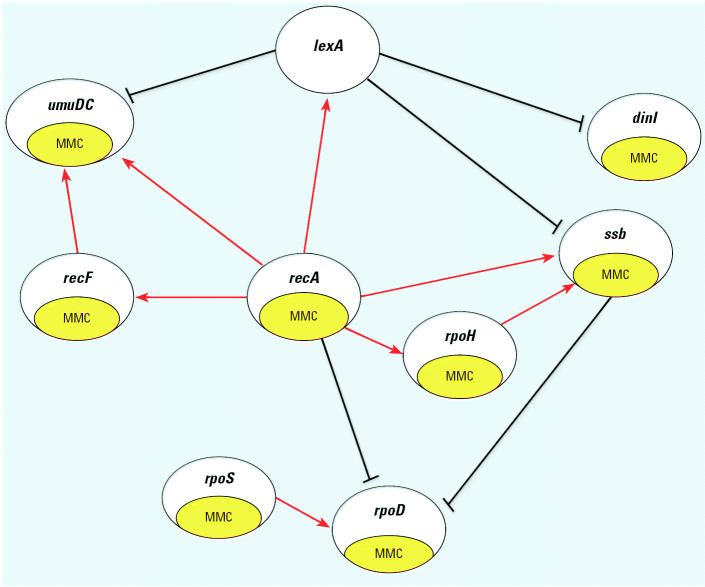
Network linkages of key genes in the SOS response in *E. coli* as identified by the TAO-Gen algorithm.

**Figure 3:**
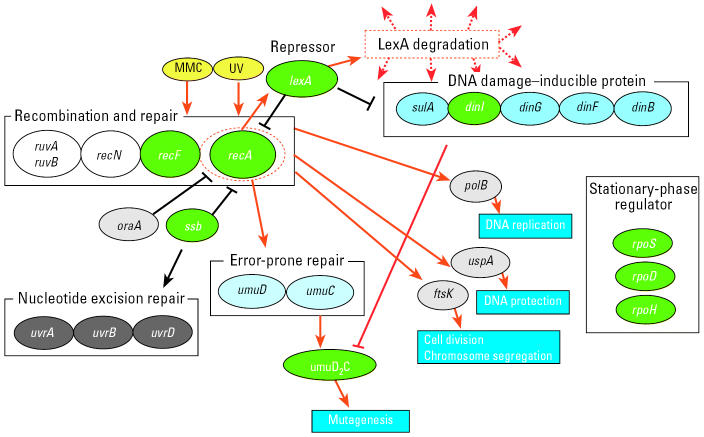
A literature-based linkage map between genes in the SOS response in *E. coli*. The map represents inducible genes/proteins in the SOS response for repair from DNA damage. Black lines indicate pathways in the normal repair process and red lines with arrows activation/induction due to an exposure to damaging agents. Recombination and repair, DNA damage–inducible protein, nucleotide excision repair, error-prone repair, and stationary-phase regulator have family molecules in each box. Green circles are genes used for the analysis.

The TAO-Gen algorithm was applied to real time PCR data on nine genes (*recA, lexA*, *ssb, recF, dinI, umuDC, rpoD, rpoH,* and *rpoS*) from the SOS pathway in *E. coli* as described above. Data consisted of 11 separate relative changes in gene expression: 9 samples for which a plasmid was inserted for one of the nine genes, a single construct for a combination of two genes (*lexA* and *recA*), and a modification of the culture (1.5 × increase in mitomycin C) in wild-type cells. Figure 2 illustrates the optimal gene interaction network identified by the TAO-Gen algorithm for these data. It is generally believed that the SOS regulon in *E. coli* is predominantly under the control of the products of the genes *lexA* and *recA*. Figure 3 illustrates a literature-based linkage map between genes in the SOS response for the repair of DNA damage. When genotoxins, such as ultraviolet radiation and MMC, damage DNA base nucleotides, the replication process is activated and a region of single-stranded DNA (ssDNA) is formed. RecA (the product of *recA*) coats ssDNA, signaling the SOS response. RecA/ssDNA stimulates degradation of LexA (the products of *lexA*), which is a repressor of RecA in the normal repair process. This inactivation of LexA affects other genes involved directly in SOS response, such as *dinI*, and downstream genes involved in DNA replication, cell division and mutagenesis, such as *rpoS* (Beuning 2004; Janion 2001; Lindner 2004; Lusetti 2002; McKenzie 2000; Rangarajan 2002). The results from the TAO-Gen algorithm are given in Figure 2 and support this role for LexA with significant repressor activity on *umuDC*, *dinI* and *ssb*. In contrast, RecA, the gene product of *recA*, is expected to serve as an activator of the SOS regulon. Figure 2 indicates that *recA* serves as a central node in the regulation of genes in the SOS pathway, showing significant activation of *lexA*, *recF*, *umuDC*, *rpoH* and *ssb* and significant repression of *rpoD*. There are four remaining significant linkages: *ssb* and *rpoS* repress and activate *rpo*D, respectively, and *recF* activates *umuDC* and *rpoH* activates *ssb*. Table 1 provides summary information on the parameter estimates estimated by treating the identified network (Figure 2) as known and quantifying the linkages between genes by the method of Toyoshiba et al. (2004). With the exception of the plasmid-induced change in *recF*, all linkages in Figure 2 are statistically significant (*p* < 0.05).

An indicator variable was used to separate data with and without plasmid insertion for each gene. For all nine genes, plasmid inserts increased mRNA levels ranging from a nonsignificant (*p* = 0.31) 1.06-fold increase for *recF* to a significant (*p* < 0.01) 28-fold increase for *rpoH*. Changes in the level of MMC had significant effects on eight of the nine genes, the sole exception being *lexA,* which did not appear to be directly affected by changes in MMC. This finding is in contrast to what was believed to be the presumed transcriptional target of MMC, *recA*. It was previously suggested that all other MMC-induced changes in transcription are mediated through *recA*. In this analysis the largest impacts of MMC on transcription were for *rpoH* and *rpoS* (an ~12.3-fold increase in activity for each doubling of the MMC level) followed by effects on *recA*, *dinI* and *umuDC* (approximately a 1.9-fold increase in activity for each doubling of MMC level).

Our best network (Figure 2) and the literature-based network (Figure 3) support the notion that the activation of the SOS system is through activation of *recA*. Increases in *recA* result in activation of *umuDC* and *ssb*, critical components in the activation of repair of single-strand DNA damage. An increase in *recA* also induces an increase in *lexA,* which serves to suppress the activity induced by *recA* in *umuDC* and *ssb*. *rpoH* appears to serve as an independent activator of *ssb* with signaling from *recA* and possibly other genes not included in the network. Finally, while *rpoS* and *rpoD* seem to be linked to the network, they appear to be under control of other genes in the network rather than exerting control over the SOS response. Recent articles hypothesized possible roles for roles for RpoS, LexA and RecA in global stress gene regulation, but clear conclusions are not yet available (Gerard et al. 1999; Gill et al. 2000).

With such a small number of samples (11) relative to the number of genes involved (9), it is likely that the resulting model is overly sensitive to any one data set. To evaluate this, we applied the TAO-Gen analysis to 11 data sets in which one sample from the original data was eliminated. Generally, removing a sample resulted in deletion of a connection rather than inclusion of new connections. Removing the *dinI* plasmid insert had no impact on the resulting network; removing the double plasmid insert only added a single additional connection between *rpoH* and *rpoS;* and removing the MMC sample (no plasmid insert) removed only one linkage (*rpoH-rpoS*). All other sample removals resulted in two to five changes in the network with no more than one additional linkage in any case. Three linkages (*recA* to *lexA*, *lexA* to *umuDC* and *recF* to *umuDC*) remained unchanged for all sample deletions; all others were simply eliminated once or twice for specific sample deletions with the exceptions of recA to rpoH, which was removed in four sample deletions, and *rpoS* to *rpoD* which was removed in one sample deletion and switched direction for three sample deletions. All additional linkages (there were six sample deletions with one additional linkage in each case) included at least one of the stationary phase regulators (*rpoH, rpoS, rpod*), suggesting the linkage between this class of genes and the SOS pathway may be too distant to quantify. Generally, with the exception of linkages to and between the stationary phase regulators, the model was fairly stable across deletions of single samples from the data set.

## Discussion

The network presented in Figure 2 is substantially smaller than that proposed by Gardner et al. (2003) Using their NIR (network identification by multiple regression) algorithm, they identified a network with 45 linkages (excluding changes due to MMC or the plasmids) compared with our network with only 13 gene linkages. There are significant differences between the NIR and TAO-Gen algorithms that directly impact affect these findings. In the NIR algorithm, parents for each gene are discovered independently of the other genes by finding the five parents that maximize the usual likelihood of the data given the model. The choice of five parents is somewhat arbitrary, and the use of the data multiple times for each gene overstates the information available. In addition each gene is allowed to be a parent of itself, creating a singularity in the model that results in most other parents having no significant impact on any given gene expression level. Of the 36 linkages (six parents were chosen for *recF*) identified by the NIR algorithm, all nine genes have significant linkages with themselves as parents. Of the remaining 27 linkages, only 9 are significant (*p* < 0.05 by a Wald test) as follows: *ssb* activates *recA* and *recF*, *recA* suppresses *lexA* and *rpoH*, *dinI* activates *recA*, *umuDC* and *rpoS*, *rpoH* suppresses *rpoD,* and *rpoS* suppresses *recF*. The TAO-Gen algorithm, in contrast, restricts the network to acyclic linkages and uses the full likelihood (all of the data simultaneously) to find the best network. Of the 9 significant linkages identified by the NIR algorithm, the TAO-Gen algorithm identified only the suppression of *lexA* and *rpoH* by *recA*. The significant findings by the NIR algorithm do not identify *recA* as a key controlling gene in the network whereas the TAO-Gen algorithm does.

Mathematically the data obtained by Gardner et al. (2003) does not have sufficient statistical support to identify a cyclical network. The data required to estimate parameters in a cyclical network must contain observations at different time points to estimate the dynamic characteristics of a cyclic network. To directly compare the Gardner et al. network to the one shown in Figure 2, the Gardner et al. network was made acyclic by removing the linkages for genes as their own parents and by removing the linkage between *dinI* and *lexA*. When the Bayesian estimation algorithm was applied (Toyoshiba et al. 2004), the posterior log-likelihood for this model had a mean value of 329.2 compared with 354.7 from the model identified by the TAO-Gen algorithm, suggesting a considerably better fit of the model in Figure 2 to the data. Using the “known model” suggested by Gardner et al. (2003), the resulting mean of the posterior log-likelihood was 311.0, also suggesting a serious lack of fit.

So is the model presented in Figure 2 a better representation of the gene interaction network for the SOS pathway in *E. coli*? The resulting network has identified the significant gene linkages seen in the data. It correctly identifies *recA* as playing the major role in control of this pathway and provides estimates of the steady-state linkage between these genes. The interpretation of the values estimated for the parameters linking genes in Figure 2 does not preclude that the network could be dynamic with substantial feedback; such a possibility is likely. But given the data available, this network identifies the key linkages that exist as the network changes from one steady-state to another. What this means can be explained by example. The activation of *recF* by *recA* has a mean value of 0.393. This implies that, if the steady-state expression of *recA* doubles, then the steady-state expression of *recF* would fold increase by the exponential of 0.393 × *ln*(2) or 1.32-fold. Singular changes in any gene in the network can easily be used to calculate new steady-state conditions for the network.

Illustrating that one can achieve a network from a given data set does not assess the reliability of a new algorithm. A better method is to evaluate the probability of choosing the correct network using data from a known network. Monte Carlo simulation was used to generate 100,000 artificial gene expression arrays from the network in Figure 1 using four different sets of model parameters as defined in Table 2. When the algorithm is applied to these data, the resulting optimal network is identical to the network shown in Figure 1 in all four cases. This illustrates that the algorithm is consistent for extremely large data sets. To assess the behavior of the algorithm for small samples, the four sets of 100,000 artificial arrays were subdivided into 1,000 data sets of 100 arrays, 2,000 data sets of 50 arrays, 4,000 data sets of 25 arrays, and 10,000 data sets of 10 arrays. For each data set, the algorithm was applied and an optimal network chosen; the results appear in Table 2.

There are 543 possible acyclic networks that can arise from a combination of four genes. Table 2 summarizes the frequency (from 543 total networks) seen for various network structures (column 3 is the correct structure). For example, with 100 arrays in the sample, the correct network is chosen 922/100 = 92% of the time for parameter set A (row 1 of Table 2). Generally, with 100 replicate arrays, the search algorithm is better than 92% effective in finding the right network. The most common error in finding an array for this sample size is to add an additional linkage between gene 2 and gene 4 (column 8 in Table 2, 1–8%). When the sample size is halved to 50 arrays, accuracy drops to between 86 and 93%, with the same additional linkage being the most common mistake (2–9%). With only 25 arrays, accuracy is still between 70 and 80%, with most of the errors occurring for the same additional linkage (4–8%), single deletions of linkages (3–4%), or reversals of individual linkages (2–3%). Replicate samples consisting of just 10 arrays surprisingly find the correct network 32–38% of the time, with 30–40% of the errors being additional linkages, single linkage removal, or single linkage reversals. The simulations suggest the algorithm generally detects networks having very close topologies to the correct one even if the sample number is severely diminished.

As noted in “Materials and Methods,” the algorithm being used to find the best network is intended as an approximation for using the posterior likelihood to identify the best network. In the last four columns of Table 2, the correct network has the best posterior likelihood in every case for which it is the optimal network. In addition the algorithm works well at placing the correct network into the top three networks, ranging from about 99% for samples involving 100 arrays to 58% for samples consisting of 10 arrays. These simulations suggest that the best directed acyclic network does not necessarily mean that all the links are real or that they are causal. Conversely, they do suggest that the limitations inherent to small sample sizes could be reduced by considering not only the best network, but several of the best networks and using other resources, such as knowledge of the existing pathways, to decide which makes the most sense.

**Figure 4:**
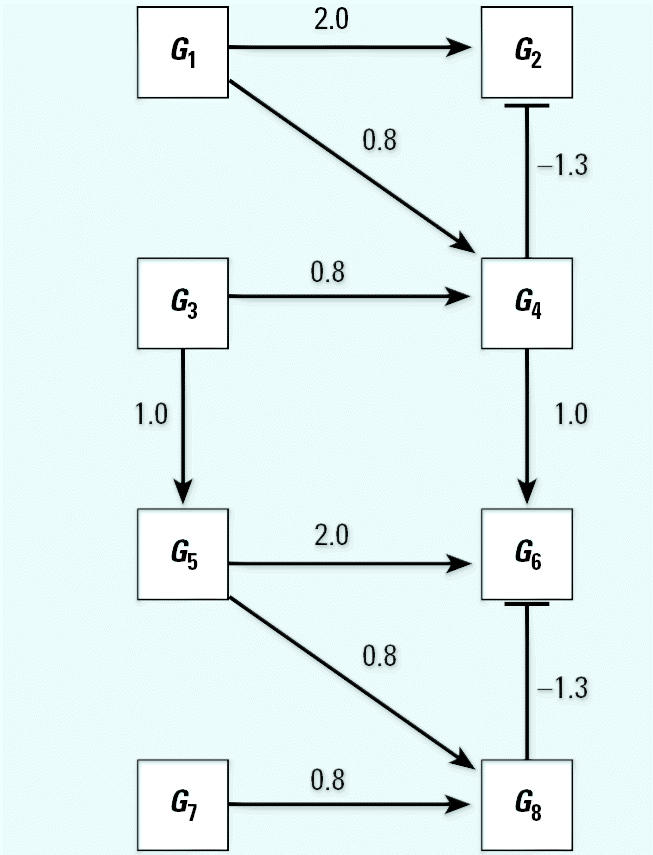
A hypothetical eight gene network used for the Monte-Carlo simulations in Table 3. The numbers attached to the arrows show linear parameters, where positive numbers correspond to up-regulations and negative numbers down-regulations.

These results were expanded to look at an eight-gene network, effectively a combination of two four-gene networks similar to that in Figure 1, where gene 2 activates gene 5 and gene 3 activates gene 8 (Figure 4). In this case it is computationally impossible to conduct the exhaustive search as in the four-gene case because the number of acyclic networks is approximately 78 × 10^13^. Instead, 1,000 data sets were randomly generated for each sample case (100, 50, 25, 10) and the TAO-Gen algorithm was applied to identify a best network for each data set. Table 3 shows the numbers of connections detected by the algorithm, where the rows and columns correspond to parents and child genes, respectively. For example, the algorithm detected the incorrect path from gene 1 to gene 2 only three times in 1,000 data sets with 100 samples. The red elements show the true connections. For 100 replicate samples (microarrays), the TAO-Gen algorithm identified the correct network in 95% of the cases. As before, the deviations from the correct model were all cases of adding an additional linkage or removing a single linkage. As the sample size dropped to 50, 25 and 10, the correct network was identified 76, 30 and 1% of the time, respectively. Even though the performance in finding the fully correct network became poor, the linkages in the correct network were generally properly identified with high frequency, again indicating that the cases where the network was incorrect generally involved single or double alterations in the pathways of the network. The simulation using eight genes accentuates the importance of study design and prior knowledge about gene linkages in trying to find the best network to explain the data.

Many issues remain to be studied. It is unclear whether the TAO-Gen algorithm works better or worse than other algorithms in identifying gene interaction networks. The main problem arises because other algorithms have not used computer simulations to examine model specifity to directly address this issue. Also, the use of acyclic models to develop gene interaction networks is somewhat limited. A fully dynamic model using time-dependent differential equations could be used with the TAO-Gen algorithm provided multitime point data were available; the method would simply need to link models across time as suggested elsewhere (Toyoshiba et al. 2004) or use dynamic Bayesian networks. Here we assume samples are independent; in time-course data, that would not necessarily be the case and the error structure between samples would need to be altered (in Equation 4 and subsequent derivations) to account for the longitudinal nature of such data. In any case the analysis would certainly require more data than are generally available. Perhaps the biggest advantage of using a Bayesian-linked analysis algorithm would occur when prior knowledge, based on known biologic linkages such as those derived from bioinformatic evaluations of transcription sequences, is used to limit the range of networks to be explored. The TAO-Gen algorithm could work in these situations but would need to be modified to use a prior different than the uniform prior used in this case.

## Conclusion

In this article we have presented the TAO-Gen algorithm for identifying gene interaction networks. The algorithm was applied to data on the SOS pathway in *E. coli* to identify gene linkages. The resulting network is shown to be superior to a network derived by the NIR algorithm in (Gardner et al. 2003) both biologically and statistically. Unlike the NIR algorithm, this algorithm identified a statistically significant role of *recA* in controlling the SOS pathway; the linkages from *recA* in the NIR-derived network were generally not significant. To demonstrate the accuracy of the algorithm for varying sample sizes, a simulation study was performed. It was found that for moderate-size networks the algorithm performs accurately, with most errors being minor additions or deletions of a single linkage. However, the simulations do suggest that sample sizes need to be increased if large networks are to be identified and quantified using gene expression data.

## Figures and Tables

**Table 1:** Estimated means, standard deviations and percentage above 0 for all interactions in SOS response genes for *E. coli* identified as linked by the TAO-Gen algorithm (see Figure 2).

From	To	Type	Mean	SD	% < 0
*recA*	*lexA*	Activate	0.435	0.065	0.00
	*ssb*	Activate	0.137	0.056	0.99
	*recF*	Activate	0.393	0.161	0.93
	*umuDC*	Activate	0.365	0.129	0.42
	*rpoD*	Repress	−0.356	0.091	99.97
	*rpoH*	Activate	0.193	0.093	2.06
*lexA*	*ssb*	Repress	−0.158	0.065	98.86
	*dinI*	Repress	−0.287	0.156	96.61
	*umuDC*	Repress	−0.550	0.169	99.85
*ssb*	*rpoD*	Repress	−0.077	0.029	99.46
*recF*	*umuDC*	Activate	0.512	0.204	0.81
*rpoH*	*ssb*	Activate	0.031	0.012	0.55
*rpoS*	*rpoD*	Activate	0.496	0.108	0.02
Plasmid insert	*recA*	Activate	0.458	0.080	0.00
	*lexA*	Activate	0.396	0.041	0.00
	*ssb*	Activate	2.443	0.039	0.00
	*recF*	Activate	0.062	0.130	30.95
	*dinI*	Activate	1.188	0.110	0.00
	*umuDC*	Activate	1.007	0.093	0.00
	*rpoD*	Activate	1.409	0.069	0.00
	*rpoH*	Activate	3.319	0.074	0.00
	*rpoS*	Activate	0.513	0.100	0.00
MMC	*recA*	Activate	0.979	0.282	0.06
	*ssb*	Activate	0.479	0.108	0.05
	*recF*	Activate	0.637	0.345	3.28
	*dinI*	Activate	0.896	0.282	0.07
	*umuDC*	Activate	0.969	0.252	0.05
	*rpoD*	Activate	0.460	0.221	2.12
	*rpoH*	Activate	1.233	0.204	0.00
	*rpoS*	Activate	1.255	0.248	0.00

**Table 2:** Results from 100,000 Monte Carlo simulations of four hypothetical four-gene networks (A, B, C, D)*^a^* describing the ability of the TAO-Gen algorithm to specify the correct network.

		Frequency (%) of resulting optimal network structure							Rank (%) of the posterior likelihood for the true network over all possible 543 acyclic networks			
Sample size	True model	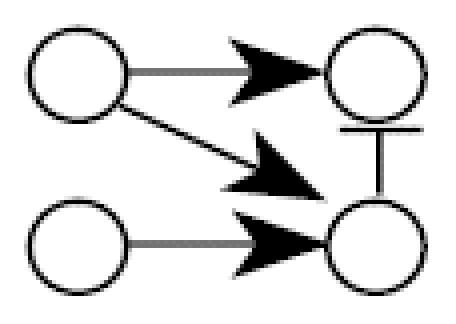	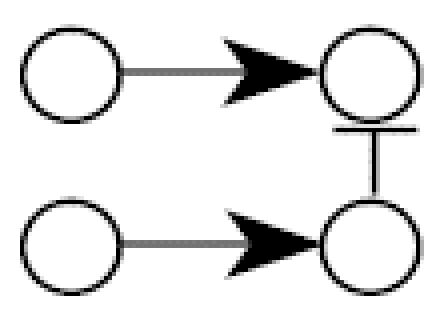	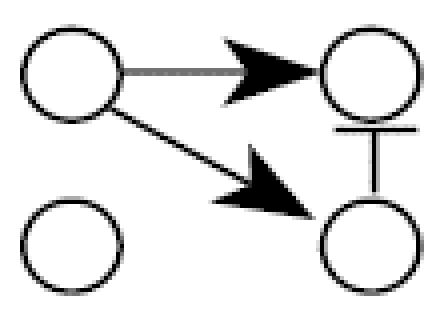	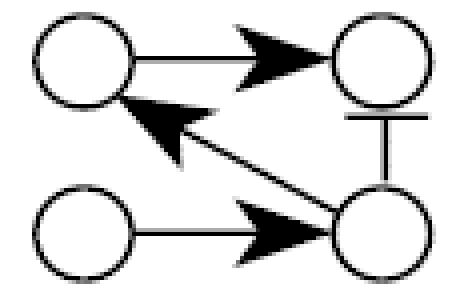	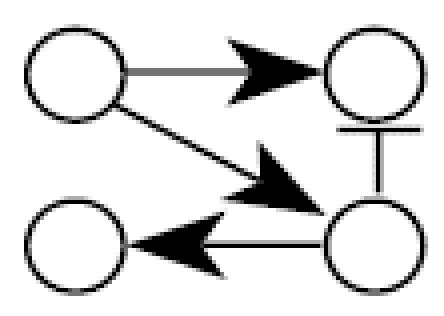	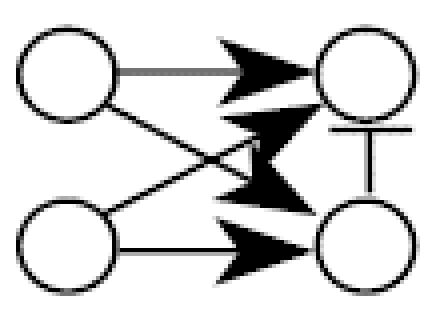	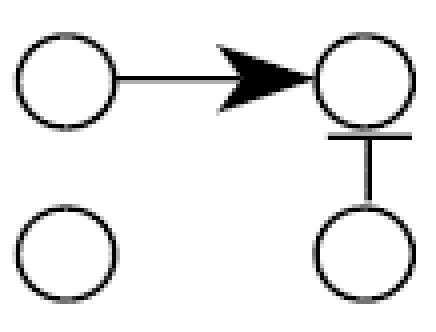	1	2	3	4–10
100 arrays	A	922 (92)	0 (0)	0 (0)	0 (0)	0 (0)	68 (7)	0 (0)	922 (92)	52 (5)	10 (1)	16 (2)
1,000 sims	B	977 (98)	0 (0)	0 (0)	0 (0)	0 (0)	6 (1)	0 (0)	977 (98)	17 (2)	4 (0.4)	2 (0.2)
	C	929 (93)	0 (0)	0 (0)	0 (0)	0 (0)	71 (7)	0 (0)	929 (93)	50 (5)	8 (1)	13 (1)
	D	980 (98)	0 (0)	0 (0)	0 (0)	0 (0)	6 (1)	0 (0)	980 (98)	13 (1)	5 (0.5)	2 (0.2)
50 arrays	A	1,716 (86)	4 (0.2)	3 (0.2)	6 (0.3)	4 (0.2)	165 (8)	0 (0)	1,716 (87)	157 (8)	34 (2)	70 (4)
2,000 sims	B	1,841 (92)	8 (0.4)	0 (0)	4 (0.2)	8 (0.4)	41 (2)	0 (0)	1,841 (92)	82 (4)	20 (1)	55 (3)
	C	1,745 (87)	6 (0.3)	4 (0.2)	3 (0.2)	6 (0.3)	175 (9)	0 (0)	1,745 (88)	128 (6)	41 (2)	62 (3)
	D	1,860 (93)	4 (0.2)	0 (0)	2 (0.1)	0 (0)	46 (2)	0 (0)	1,860 (93)	68 (3)	30 (2)	42 (2)
25 arrays	A	2,920 (73)	76 (2)	72 (2)	56 (1)	77 (2)	328 (8)	3 (0.1)	2,920 (73)	423 (10)	112 (3)	387 (10)
4,000 sims	B	3,179 (80)	92 (2)	55 (1)	48 (1)	47 (1)	192 (5)	8 (0.2)	3,179 (79)	348 (9)	133 (3)	249 (6)
	C	2,891 (72)	60 (1)	100 (2)	56 (1)	76 (2)	296 (7)	4 (0.1)	2,891 (72)	404 (10)	114 (3)	444 (11)
	D	3,086 (77)	76 (2)	96 (2)	48 (1)	48 (1)	164 (4)	8 (0.2)	3,086 (77)	328 (8)	149 (4)	365 (9)
10 arrays	A	3,198 (32)	909 (9)	741 (7)	230 (2)	149 (2)	328 (3)	497 (5)	3,198 (32)	1,027 (10)	781 (8)	2,389 (24)
10,000 sims	B	3,768 (38)	1,002 (10)	1,051 (10)	220 (2)	309 (3)	378 (4)	567 (6)	3,768 (38)	966 (10)	821 (8)	2,519 (25)
	C	3,177 (32)	892 (9)	691 (7)	230 (2)	151 (2)	398 (4)	457 (5)	3,177 (32)	1,232 (12)	769 (8)	2,347 (23)
	D	3,768 (38)	1,052 (10)	1,031 (10)	280 (3)	259 (3)	538 (5)	477 (5)	3,768 (38)	1,146 (11)	871 (9)	2,371 (24)

a (A) β_14_ = 2.0, β_13_ = 0.8, β_23_ = 0.8, β_34_ = −1.3, σ_1_ = σ_2_ = σ_3_ = σ_4_ = 1.0 (B) β_14_ = 2.0, β_13_ = 0.8, β_23_ = 0.8, β_34_ = −5.0, σ_1_ = σ_2_ = σ_3_ = σ_4_ = 1.0 (C) β_14_ = 2.0, β_13_ = 0.8, β_23_ = 0.8, β_34_ = −1.3, σ_1_ = σ_2_ = σ_3_ = σ_4_ = 1/3 (D) β_14_ = 2.0, β_13_ = 0.8, β_23_ = 0.8, β_34_ = −5.0, σ_1_ = σ_2_ = σ_3_ = σ_4_ = 1/3
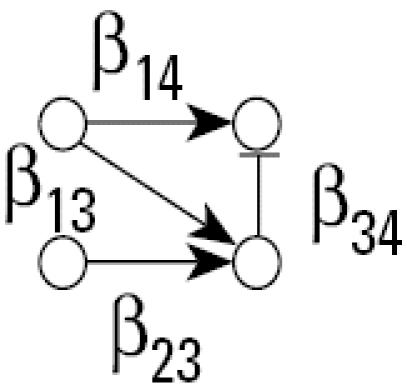

**Table 3:** Number (percent) of linkages between two genes identified by the TAO-Gen algorithm in 1,000 Monte Carlo simulations of the hypothetical eight-gene network shown in Figure 3.

	From gene number	To cell number							
		1	2	3	4	5	6	7	8
100 Chips	1	——	3 (0.3)	1,000 (100)*^a^*	1,000 (100)*^a^*	4 (0.4)	1 (0.1)	4 (0.4)	5 (0.5)
	2	0 (0)	——	999 (99.9)*^a^*	9 (0.9)	1,000 (100)*^a^*	1 (0.1)	3 (0.3)	7 (0.7)
	3	0 (0)	1 (0.1)	——	1,000 (100)*^a^*	0 (0)	0 (0)	0 (0)	1,000 (100)*^a^*
	4	0 (0)	0 (0)	0 (0)	——	0 (0)	0 (0)	0 (0)	0 (0)
	5	0 (0)	0 (0)	0 (0)	3 (0.3)	——	0 (0)	1,000 (100)*^a^*	999 (99.9)*^a^*
	6	2 (0)	0 (0)	2 (0.2)	2 (0.2)	2 (0.2)	——	1,000 (100)*^a^*	8 (0.8)
	7	0 (0)	0 (0)	0 (0)	1 (0.1)	0 (0)	0 (0)	——	1,000 (100)*^a^*
	8	0 (0)	0 (0)	0 (0)	0 (0)	0 (0)	0 (0)	0 (0)	——
50 Chips	1	——	4 (0.4)	980 (98)*^a^*	1,000 (100)*^a^*	23 (2.3)	11 (1.1)	23 (2.3)	8 (0.8)
	2	8 (0.8)	——	977 (97.7)*^a^*	19 (1.9)	989 (98.9)*^a^*	6 (0.6)	13 (1.3)	24 (2.4)
	3	14 (1.4)	2 (0.2)	——	995 (99.5)*^a^*	3 (0.3)	3 (0.3)	9 (0.9)	1,000 (100)*^a^*
	4	0 (0)	0 (0)	5 (0.5)	——	0 (0)	0 (0)	1 (0.1)	0 (0)
	5	2 (0.2)	9 (0.9)	14 (1.4)	7 (0.7)	——	4 (0.4)	991 (99.1)*^a^*	973 (97.3)*^a^*
	6	10 (1)	4 (0.4)	15 (1.5)	13 (1.3)	15 (1.5)	——	989 (98.9)*^a^*	11 (1.1)
	7	1 (0.1)	0 (0)	0 (0)	7 (0.7)	7 (0.7)	2 (0.2)	——	998 (99.8)*^a^*
	8	0 (0)	0 (0)	0 (0)	5 (0.5)	0 (0)	0 (0)	2 (0.2)	——
25 Chips	1	——	33 (3.3)	832 (83.2)*^a^*	960 (96)*^a^*	26 (2.6)	18 (1.8)	26 (2.6)	50 (5)
	2	20 (2)	——	751 (75.1)*^a^*	63 (6.3)	912 (91.2)*^a^*	14 (1.4)	57 (5.7)	94 (9.4)
	3	37 (3.7)	46 (4.6)	——	933 (93.3)*^a^*	10 (1)	5 (0.5)	46 (4.6)	962 (96.2)
	4	1 (0.1)	0 (0)	63 (6.3)	——	2 (0.2)	0 (0)	2 (0.2)	11 (1.1)
	5	5 (0.5)	50 (5)	59 (5.9)	34 (3.4)	——	9 (0.9)	905 (90.5)*^a^*	811 (81.1)
	6	9 (0.9)	10 (1)	19 (1.9)	38 (3.8)	64 (6.4)	——	857 (85.7)*^a^*	69 (6.9)
	7	2 (0.2)	0 (0)	21 (2.1)	24 (2.4)	60 (6)	19 (1.9)	——	964 (96.4)
	8	2 (0.2)	0 (0)	13 (1.3)	9 (0.9)	0 (0)	0 (0)	33 (3.3)	——
10 Chips	1	——	51 (5.1)	516 (51.6)*^a^*	702 (70.2)*^a^*	63 (6.3)	30 (3)	73 (7.3)	141 (14.1)
	2	49 (4.9)	——	335 (33.5)*^a^*	155 (15.5)	590 (59)*^a^*	35 (3.5)	171 (17.1)	166 (16.6)
	3	73 (7.3)	84 (8.4)	——	596 (59.6)*^a^*	67 (6.7)	16 (1.6)	126 (12.6)	641 (64.1)*^a^*
	4	23 (2.3)	15 (1.5)	227 (22.7)	——	11 (1.1)	8 (0.8)	22 (2.2)	71 (7.1)
	5	16 (1.6)	106 (10.6)	79 (7.9)	87 (8.7)	——	33 (3.3)	519 (51.9)*^a^*	375 (37.5)*^a^*
	6	35 (3.5)	30 (3)	73 (7.3)	93 (9.3)	95 (9.5)	——	408 (40.8)*^a^*	187 (18.7)
	7	9 (0.9)	18 (1.8)	74 (7.4)	79 (7.9)	168 (16.8)	51 (5.1)	——	693 (69.3)*^a^*
	8	3 (0.3)	2 (0.2)	68 (6.8)	51 (5.1)	24 (2.4)	8 (0.8)	135 (13.5)	——

a Linkage that exists in the original simulated model.
